# Outcome of different post-orchiectomy management for stage I seminoma: Japanese multi-institutional study including 425 patients

**DOI:** 10.1111/j.1442-2042.2010.02645.x

**Published:** 2010-10-18

**Authors:** Tomomi Kamba, Toshiyuki Kamoto, Kazutoshi Okubo, Satoshi Teramukai, Yoshiyuki Kakehi, Tadashi Matsuda, Osamu Ogawa

**Affiliations:** 1 Department of Urology, Kyoto University Graduate School of MedicineKyoto; 3 Translational Research Center, Kyoto University HospitalKyoto; 2 Department of Urology, Faculty of Medicine, Miyazaki UniversityMiyazaki; 4 Department of Urology, Faculty of Medicine, Kagawa UniversityKagawa; 5 Department of Urology, Kansai Medical UniversityHirakata, Osaka, Japan

**Keywords:** chemotherapy, outcome, radiotherapy, stage I seminoma, surveillance

## Abstract

**Objectives:**

To clarify the contemporary clinical outcome of stage I seminoma and to provide information on treatment options to patients.

**Methods:**

A retrospective analysis of 425 patients who underwent orchiectomy for stage I seminoma between 1985 and 2006 at 25 hospitals in Japan. Relapse-free survival rates were calculated using the Kaplan–Meier method and clinicopathological factors associated with relapse were examined by univariate and multivariate analyses using the Cox proportional hazards model.

**Results:**

A total of 30 out of 425 patients had relapsed. Relapse-free survival rates at 10 years were 79, 94 and 94% in the surveillance, chemotherapy and radiotherapy groups, respectively. Post-orchiectomy management and rete testis invasion were identified as independent predictive factors associated with relapse. Rete testis invasion remained to be an independent predictive factor, even if the cases with relapses in the contralateral testis were censored. Only one patient, who relapsed after adjuvant radiotherapy, died of the disease. Overall survival at 10 years was 100, 100 and 99% in the surveillance, chemotherapy and radiotherapy groups, respectively. More than half of the patients were lost to follow up within 5 years.

**Conclusions:**

The outcome of Japanese patients with stage I seminoma is similar to previously published Western reports. Surveillance policy is becoming a popular option in Japan, although the relapse rate in patients opting for surveillance policy is higher than those opting for adjuvant chemotherapy or radiotherapy. Rete testis invasion is an independent predictive factor associated with relapse regardless of the post-orchiectomy management. Long-term follow up is mandatory for detection of late relapse.

## Introduction

Approximately 75% of seminoma patients present with stage I disease.^1^ After orchiectomy, stage I seminoma patients can be managed by surveillance, adjuvant radiotherapy or adjuvant chemotherapy. Almost all patients can be salvaged, even after a relapse, mainly by chemotherapy; the overall cure rates approach 100%, regardless of the post-orchiectomy management.^2^,^3^

Because the incidence of testicular germ cell tumors has been rising in Japan,^4^,^5^ even though it is still lower than that in Western countries, we should pay more attention to this disease. However, little is known about post-orchiectomy management patterns and predictive factors associated with relapse in the Japanese population with stage I seminoma.^6^–^8^ In the present study, we carried out a retrospective multi-institutional survey to establish the clinical outcome and predictive factors for relapse of stage I seminoma, and to provide information on treatment options to patients.

## Methods

We registered 425 testicular cancer (TC) patients diagnosed with stage I seminoma who underwent radical orchiectomy between 1985 to 2006 at 25 Japanese institutions, including three university hospitals. We collected clinical and pathological data from the medical records including age at orchiectomy and pathological information, such as pT stage, tumor size, presence or absence of anaplastic feature, syncytiotrophoblastic cells, lymphovascular invasion, rete testis invasion or spermatic cord invasion. We also gathered clinical information on serum tumor markers at initial diagnosis, post-orchiectomy management, relapse pattern, death and the patient's follow-up schedule. The significance of difference in the age at orchiectomy was assessed using anova. The significance of differences in the distribution of clinicopathological characteristics or relapse pattern among the post-orchiectomy management groups was determined by Pearson's χ^2^-test or Fisher's exact test, respectively.

The primary end-point was relapse-free survival (RFS) calculated from the date of orchiectomy to that of a diagnosis of any relapse, including a relapse in the contralateral testis, death from any cause or last follow up. To estimate the hazard ratio (HR) and to identify the predictive factors associated with RFS, we carried out univariate and multivariate analyses with backward elimination using the Cox proportional hazards model. A two-sided *P* < 0.05 was regarded as statistically significant.

## Results

### Patient characteristics

The characteristics of 425 patients with stage I seminoma are presented in Table 1. Surveillance policy was provided to 186 patients, adjuvant chemotherapy to 57 and adjuvant radiotherapy to 182 as post-orchiectomy management. The median age at orchiectomy was higher in the adjuvant chemotherapy group (40 years) than in the surveillance (36 years) and adjuvant radiotherapy (36 years) groups, but the difference was not statistically significant. The median follow up duration of the entire study group was 52.5 months (range 0.1–248.5 months); it was shorter in the surveillance group (44.9 months) than in the adjuvant chemotherapy (58.4 months) and radiotherapy group (60.8 months).

**Table 1:** Patient characteristics

	Total	Post-orchiectomy management			P*
		Surveillance	Chemotherapy	Radiation	
No. patients	425	186	57	182	
Median age at orchiectomy, year (range)	36 (19–84)	36 (19–84)	40 (24–66)	36 (22–64)	0.084
Median follow up, months (range)	52.5 (0.1–248.5)	44.9 (0.1–218.7)	58.4 (2.5–205.6)	60.8 (0.9–248.5)	
Post-orchiectomy management pattern by the year of orchiectomy					
1985–1989	47	4 (8.5%)	1 (2.1%)	42 (89.4%)	
1990–1994	63	21 (33.3%)	8 (12.7%)	34 (54.0%)	
1995–1999	109	39 (35.8%)	16 (14.7%)	54 (49.5%)	
2000–2006	206	122 (59.2%)	32 (15.5%)	52 (25.2%)	

* anova.

Until 1990, 89.4% of TC patients were treated with adjuvant radiotherapy after orchiectomy. However, the proportion continued to decrease, resulting in 25.2% in the 2000s. Meanwhile, 8.5% of the patients were managed with a surveillance policy before 1990, but the proportion increased to 59.2% in the 2000s. Before 1990, only one patient was treated with adjuvant chemotherapy. In the 1990s, the proportion of patients treated with adjuvant chemotherapy was increasing and it reached 15.5% in the 2000s.

Regimens of adjuvant chemotherapy were single-agent carboplatin in 51 patients (89.5%); etoposide and cisplatin in one patient (1.8%), bleomycin, etoposide and cisplatin in one patient (1.8%), and cisplatin, vinblastine and bleomycin, or vinblastine, actinomycin-D and bleomycin in four patients (7.0%). The fields of irradiation in adjuvant radiotherapy were para-aorta and ipsilateral pelvis in 130 patients (71.4%), para-aorta and bilateral pelvis in 18 patients (9.9%), para-aorta alone in 11 patients (6.0%) and unspecified in 23 patients (12.6%).

The distribution of clinicopathological characteristics according to post-orchiectomy management is listed in Table 2. The proportion of the patients with pT1 seminoma was significantly higher in the surveillance group (85.8%) than in the chemotherapy (63.8%) or radiotherapy group (76.2%). The patients with anaplastic seminoma or spermatic cord invasion were more frequently treated with adjuvant chemotherapy (10.9, 10.6%) or radiotherapy (6.3, 6.5%) than by surveillance (2.5, 1.9%). The distribution of other factors was similar among the three groups.

**Table 2:** Distribution of clinicopathological characteristics

Factor	Post-orchiectomy management			P*
	Surveillance	Chemotherapy	Radiation	
Tumor size				
<5 cm	67 (56.3%)	14 (38.9%)	39 (44.8%)	0.101
≥5 cm	52 (43.7%)	22 (61.1%)	48 (55.2%)	
Unknown	67	21	95	
pT stage				
pT1	133 (85.8%)	30 (63.8%)	109 (76.2%)	0.003
≥pT2	22 (14.2%)	17 (36.2%)	34 (23.8%)	
Unknown	31	10	39	
Anaplastic seminoma				
Yes	4 (2.5%)	5 (10.9%)	10 (6.3%)	0.052
No	159 (97.5%)	41 (89.1%)	148 (93.7%)	
Unknown	23	11	24	
Syncytiotrophoblastic cell				
Yes	10 (7.2%)	2 (5.4%)	6 (6.0%)	0.894
No	129 (92.8%)	35 (94.6%)	94 (94.0%)	
Unknown	47	20	82	
Lymphovascular invasion				
Yes	12 (8.3%)	6 (16.7%)	19 (17.1%)	0.085
No	132 (91.7%)	30 (83.3%)	92 (82.9%)	
Unknown	42	21	71	
Rete testis invasion				
Yes	12 (9.0%)	4 (10.5%)	5 (5.0%)	0.418
No	122 (91.0%)	34 (89.5%)	95 (95.0%)	
Unknown	52	19	82	
Spermatic cord invasion				
Yes	3 (1.9%)	5 (10.6%)	10 (6.5%)	0.027
No	157 (98.1%)	42 (89.4%)	143 (93.5%)	
Unknown	26	10	29	
Elevation of serum LDH				
Yes	63 (33.9%)	18 (31.6%)	61 (33.5%)	0.949
No	123 (66.1%)	39 (68.4%)	121 (66.5%)	
Elevation of serum HCG				
Yes	64 (34.4%)	28 (49.1%)	73 (40.1%)	0.123
No	122 (65.6%)	29 (50.9%)	109 (59.9%)	
Elevation of LDH and/or HCG				
Yes	80 (43.0%)	19 (33.3%)	68 (37.4%)	0.331
No	106 (57.0%)	38 (66.7%)	114 (62.6%)	

* Pearson's chi-square test. HCG, human chorionic gonadotropin; LDH, lactate dehydrogenase.

### Pattern of relapse and treatment on relapse

During the follow up duration, 30 patients experienced a relapse of the disease, including 19 patients in the surveillance group, two patients in the chemotherapy group and nine patients in the radiotherapy group. The pattern of relapse and treatment on relapse in these patients is summarized in Table 3.

**Table 3:** Pattern of relapse and treatment at relapse

	Post-orchiectomy management			P*
	Surveillance	Chemotherapy	Radiation	
No. patients relapsed	19	2	9	
Median time to relapse, months (range)	21.0 (2.5–101.3)	42.8 (29.8–55.8)	37.9 (3.8–173.1)	
Time to relapse (no. patients, %)				
0–2 years	12 (63.2%)	0	4 (44.4%)	
2–5 years	3 (15.8%)	2 (100%)	3 (33.3%)	
5–10 years	4 (21.1%)	0	1 (11.1%)	
Over 10 years	0	0	1 (11.1%)	
Site of relapse (no. patients, %)				
Retroperitoneum	15 (78.9%)	2 (100%)	1 (11.1%)	0.001
Lung	1 (5.3%)	0	1 (11.1%)	1.000
Mediastinum	0	0	5 (55.6%)	0.002
Contralateral testis	4 (21.1%)	0	2 (22.2%)	1.000
Unknown	0	0	1 (11.1%)	0.367
2^nd^-line treatment at relapse (no. patients, %)				
Chemotherapy	13 (68.4%)	2 (100%)	7 (77.8%)	
Radiation	1 (5.3%)	0	0	
Chemotherapy + radiation	3 (15.8%)	0	0	
Orchiectomy alone	2 (10.5%)	0	0	
Unknown	0	0	2 (22.2%)	
Death during follow up (no. patients, %)				
Death of the disease	0	0	1 (11.1%)	
Death of other cause	0	0	0	

* Fisher's exact test.

The median time for relapse after orchiectomy was 21.0 months in the surveillance group, 42.8 months in the chemotherapy group and 37.9 months in the radiotherapy group. A total of 24 out of 30 relapses (80%) were diagnosed within 5 years from orchiectomy. Late relapse was observed more than 5 years after orchiectomy in four patients (21.1%) in the surveillance group and two patients (22.2%) in the radiotherapy group.

The retroperitoneum was the main site of relapse in the surveillance group (78.9%) and the chemotherapy group (100%), whereas the mediastinum and lung were the predominant sites of relapse in the radiotherapy group (66.7%). A total of 22 out of 30 patients (73.3%) with relapse were treated with chemotherapy alone, with 13 patients in the surveillance group, two in the chemotherapy group and seven in the radiotherapy group. One (3.3%) and three (10.0%) in the surveillance group were treated with radiotherapy alone and with both chemotherapy and radiotherapy, respectively, for the relapses in the retroperitonuem. Only one patient, who relapsed after adjuvant radiotherapy, died of the disease. Consequently, overall survival at 10 years was 100, 100 and 99.4% in the surveillance, chemotherapy and radiotherapy groups, respectively.

### Relapse-free survival and predictive factors of relapse

**Fig. 1:**
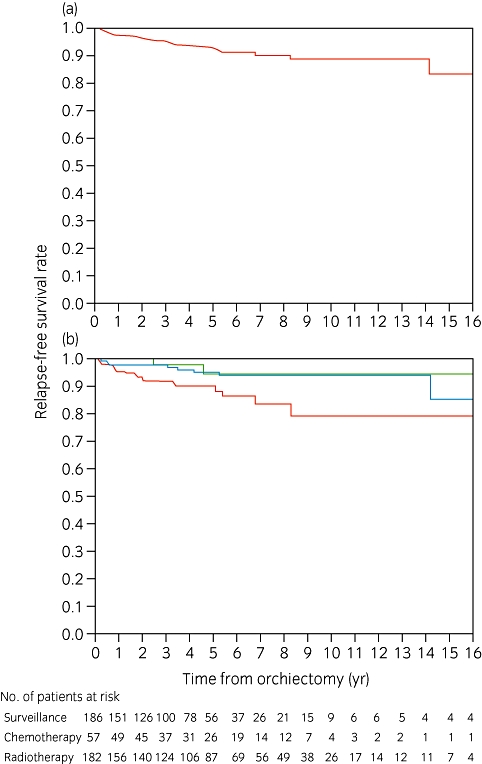
(a) Relapse-free survival of 425 patients with stage I seminoma. (b) Relapse-free survival of patients managed with surveillance, chemotherapy or radiotherapy after orchiectomy. 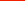, Surveillance; 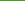, chemotherapy; 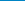, radiotherapy.

RFS for 425 patients at 5 and 10 years was 93 and 89%, respectively (Fig. 1a). RFS was significantly better in the chemotherapy and radiotherapy groups than in the surveillance group (*P* = 0.0201, Fig. 1b). RFS in the surveillance, chemotherapy and radiotherapy group was 90, 94 and 95% at 5 years and 79, 94 and 94% at 10 years, respectively.

We carried out univariate and multivariate analyses using various factors (Table 4). As a result, the post-orchiectomy management (HR: 0.31 for chemotherapy, *P* = 0.119, HR: 0.40 for radiotherapy, *P* = 0.027, global *P* = 0.043) and rete testis invasion (HR: 4.39, *P* = 0.010) were identified as independent predictive factors of relapse. Because a relapse in the contralateral testis is generally considered as a second malignancy rather than a metastasis from original testicular cancer, we carried out the same analyses by censoring the cases with relapses in the contralateral testis. Rete testis invasion was still identified as an independent predictive factor (HR: 5.83, 95% confidence intervals: 1.83–18.60, *P* = 0.003). Subgroup analysis in the surveillance group alone could not identify any predictive factors of RFS (data not shown).

**Table 4:** Univariate and multivariate analyses for relapse-free survival

Factor	Category	No. patients	No. relapse	Univariate			Multivariate		
				Hazard ratio	95% CI	P*	Hazard ratio	95% CI	P*
Age at orchiectomy	≤36 years	215	20	1					
	>36 years	206	10	0.52	0.24–1.10	0.088			
Post-orchiectomy management	Surveillance	186	19	1		0.028**	1		0.043**
	Chemotherapy	57	2	0.29	0.07–1.26	0.098	0.31	0.07–1.35	0.119
	Radiation	182	9	0.38	0.17–0.85	0.018	0.40	0.18–0.90	0.027
Tumor size	≤5 cm	120	5	1					
	>5 cm	122	10	1.93	0.66–5.65	0.232			
Elevation of LDH and/or HCG	No	167	8	1					
	Yes	258	22	1.83	0.82–4.12	0.142			
Elevation of LDH	No	283	18	1					
	Yes	142	12	1.42	0.68–2.94	0.349			
Elevation of HCG	No	260	17	1					
	Yes	165	13	1.24	0.60–2.55	0.564			
pT stage	pT1	272	18	1					
	≥pT2	73	4	0.85	0.29–2.51	0.765			
Anaplastic seminoma	No	348	21	1					
	Yes	19	3	2.17	0.65–7.29	0.211			
Syncytiotrophoblastic cell	No	258	17	1					
	Yes	18	2	2.09	0.48–9.13	0.327			
Lymphovascular invasion	No	254	17	1					
	Yes	37	3	1.17	0.34–4.01	0.798			
Rete testis invasion	No	251	14	1			1		
	Yes	21	4	5.44	1.73–17.1	0.004	4.39	1.42–13.6	0.010
Spermatic cord invasion	No	342	22	1					
	Yes	18	2	1.59	0.37–6.78	0.530			

* Wald test based on Cox proportional hazard model.

** *P*-values for global association. HCG, human chorionic gonadotropin; LDH, lactate dehydrogenase.

## Discussion

Epidemiological evidence shows a clear trend toward a worldwide increase in the incidence of TC during the past three decades; this is also the case in Japan.^5^ Meanwhile, substantial differences in the incidence and trends have been observed between geographical areas, as well as between ethnic groups. TC incidence is lowest in Asia and Africa compared with most Western countries. These differences suggest a potential role of genetic, nutritional, sociological or environmental factors in TC development.^5^ These facts encouraged us to investigate the differences, if any, in the treatment outcomes for TC between Japan and the Western countries.

In this regard, we sought to elucidate contemporary outcomes for Japanese patients with stage I seminoma treated with surveillance, adjuvant chemotherapy or adjuvant radiotherapy. Although the median follow up duration in the present study is relatively short, the results of RFS or the relapse pattern after each type of the post-orchiectomy management are equivalent to those found in previous studies,^1^,^3^,^9^–^12^ showing that the behavior of localized testicular seminoma is not different between Japanese and Western populations.

If we can predict the patients at high risk of relapse, adjuvant therapies, such as chemotherapy or radiotherapy, could be applied to these selected patients. Therefore, we sought to identify factors affecting RFS of the patients with stage I seminoma. Multivariate analysis in the entire group shows that the post-orchiectomy management and rete testis invasion are independent predictive factors of RFS. Thus, we suggest that patients with rete testis invasion should be carefully monitored regardless of the post-orchiectomy management. A large pooled analysis of patients with stage I seminoma managed with surveillance, reported tumor size and rete testis invasion as factors prognostic of relapse.^13^ Furthermore, a risk-adapted management strategy has been reported, with surveillance reserved for low-risk patients and adjuvant therapy for intermediate and high-risk patients.^14^ Unfortunately, we could not identify any high-risk group in the patients managed with surveillance, probably because of our relatively small sample size.

The present study shows a shift of the post-orchiectomy management pattern in Japanese patients. Until 1990, adjuvant radiotherapy was the predominant post-orchietomy treatment. During the 1990s, the proportion of the patients treated with a surveillance policy or adjuvant chemotherapy was increasing. In the 2000s, a surveillance policy was provided to more than half of the TC patients. This change in the management pattern might reflect on the positive and negative aspects of each treatment. Adjuvant radiotherapy has also been associated with late toxicities, such as impaired fertility, development of second malignancies and cardiovascular disease, despite its excellent cure rates.^15^ Recently, single-agent carboplatin has been recognized as a potential option for the post-orchiectomy management of stage I seminoma with encouraging short-term results,^12^,^16^,^17^ whereas long-term results on cure rates and toxicities are yet to be clarified.^18^ The surveillance policy was introduced in 1983^19^ and has become an accepted option for the management of stage I seminoma, because subsequent prospective studies consistently showed that 80–85% of patients are cured by orchiectomy alone, and virtually all patients with relapse can be salvaged by subsequent chemotherapy, minimizing the burden of treatment.^3^,^10^ The recent increase in choosing the surveillance policy in Japan might also be associated with our medical environment. First, we can easily access big medical centers, such as university hospitals, usually within an hour from our residence, which makes follow up convenient for physicians and patients during surveillance. Second, our medical costs are fully covered by public insurance. This encourages us to undergo high-tech imaging studies, such as multidetector row computed tomography (CT) or ^18^F-fluorodeoxy glucose-positron emission tomography to detect disease relapse at an early stage. Interestingly, the present study showed that the patients treated with adjuvant therapy also accepted a rigorous check-up schedule, similar to the patients in a surveillance policy (Table 5). Thus, regarding medical costs, there was no advantage that the patients with adjuvant therapies had over those with surveillance policy. This also might partly explain the reasons for their choice of surveillance policy. During the study period between 1985 and 2006, the sensitivity of CT for detection of smaller lymph nodes has been undoubtedly improved as a result of the introduction of multislice CT. It might improve the accuracy of staging in patients with stage I seminoma, which might result in the recent increase in the choice of surveillance policy. However, we should recognize that the microscopic deposits of tumor in normal-sized nodes and the distinction between tumoral and inflammatory adenopathy are beyond the scope of CT and false-negative examinations are therefore inevitable.^20^ In the present study, we cannot draw any definitive conclusion on this issue, because CT devices were not uniform among the hospitals.

**Table 5:** Post-treatment work-up interval

Follow up timing	Modality	Median work-up interval, months (range)		
		Surveillance	Chemotherapy	Radiation
Up to 2 years	Tumor marker	3 (1–12)	3 (1–6)	3 (1–6)
	Chest X-ray	3 (1–12)	3 (1–6)	3 (1–12)
	Chest CT	4 (3–12)	6 (2–12)	6 (2–12)
	Abdominal CT	4 (1–12)	4.5 (2–12)	6 (2–12)
Up to 5 years	Tumor marker	6 (2–12)	6 (1–6)	6 (2–12)
	Chest X-ray	6 (3–12)	6 (2–12)	6 (3–12)
	Chest CT	6 (3–12)	6 (3–12)	6 (3–12)
	Abdominal CT	6 (3–12)	6 (3–12)	6 (3–12)
Up to 10 years	Tumor marker	12 (4–12)	12 (2–12)	6 (3–12)
	Chest X-ray	12 (6–12)	12 (6–12)	12 (4–12)
	Chest CT	12 (6–12)	12 (6–12)	12 (6–12)
	Abdominal CT	12 (6–12)	12 (6–12)	12 (6–12)

CT, computed tomography.

The present study unexpectedly disclosed a potential problem in the management of patients with stage I seminoma in Japan. In all, 240 of 425 patients (56.5%) were lost to follow up at the entry of the present study, with a mean follow up of approximately 5 years. Surprisingly, 47.3% (88/186) of the patients managed with surveillance were lost to follow up, with a mean follow up of approximately 4.5 years. Because of the easy accessibility to hospitals in Japan and the compliant nature of the Japanese patients toward physicians, we have long believed that most TC patients are routinely followed up for the long term. As we did not plan to include the reasons for discontinuing follow up in this survey, we cannot find any definitive causes for loss-to-follow up. However, it is noteworthy that half of the patients in the present study were in the age of 35 years or younger. We speculate that young adults in this age group could not afford medical costs during their follow up, because their incomes are usually low, even if their medical costs are fully covered by insurance.^21^ Another speculation is that these young adults might have more opportunities to move to other cities for their college education or for better employment prospects. Guidelines recommend a yearly follow up for the long term, up to 10 years as a minimum follow up duration, not only for post-orchiectomy surveillance, but also for radiotherapy or chemotherapy, because late relapses have been observed to occur.^22^,^23^ Although late relapses are more frequently observed during surveillance than after adjuvant chemotherapy or radiotherapy,^24^ a surveillance policy in stage I seminoma is highly effective in a motivated center, providing excellent long-term survival with minimal morbidity and good cost-effectiveness.^25^ We should keep in mind that a high level of motivation of both patients and physicians is required for effective long-term follow up when we choose surveillance policy in the management of patients with stage I seminoma. In addition, we should also try to minimize the number of CT studies during the follow up, even in the surveillance policy if possible, particularly in younger individuals, because the cumulative radiation exposure associated with multiple CT studies has the potential for causing secondary malignancies in long-term survivors.^26^,^27^ In this context, the follow-up schedule described in EAU guidelines can be considered as a minimum requirement, which recommends abdominopelvic CT scans twice a year in the first 2 years and once a year thereafter up to 10 years.^22^

The limitations of the present study are its retrospective nature and the relatively short median follow up, which might lower the quality of analysis. However, the results obtained in the present study are comparable to those in previously published reports.^1^,^3^,^9^–^12^ We should carry out a prospective analysis on the long-term outcome of patients with stage I seminoma, and we believe that the present study provides not only baseline data for a prospective study, but also information for the patients in choosing the post-orchiectomy management.

In conclusion, the present study is the first large-scale retrospective clinical study of TC in Japan, and shows that the outcome of Japanese patients with stage I seminoma is similar to the outcome in previously published reports in Western countries. Surveillance policy is becoming a popular option in Japan, although the relapse rate in patients opting for surveillance policy is somewhat higher than in those opting for adjuvant chemotherapy or radiotherapy. Rete testis invasion is an independent predictive factor associated with relapse regardless of the post-orchiectomy management; patients with rete testis invasion should be carefully monitored for relapse. Long-term monitoring of the patients up to at least 10 years is mandatory for the detection of late relapse.
